# Role of melatonin in intestinal mucosal injury induced by restraint stress in mice

**DOI:** 10.1080/13880209.2020.1750659

**Published:** 2020-04-16

**Authors:** Rutao Lin, Zixu Wang, Jing Cao, Ting Gao, Yulan Dong, Yaoxing Chen

**Affiliations:** Beijing Advanced Innovation Center for Food Nutrition and Human Health, College of Veterinary Medicine, China Agricultural University, Haidian, China

**Keywords:** Gut, tight junction protein, antioxidant ability, autophagy, oxidative stress

## Abstract

**Context:**

A growing body of evidence demonstrates that gastrointestinal motility disorder (GIMD) and gastric stress ulcers can be induced by restraint stress, while melatonin (MT) elicits anti-inflammation and antioxidant effects.

**Objective:**

The present study investigated the mechanisms of MT-mediated protection effects on restraint stress-induced GIMD.

**Materials and methods:**

144 8-week-old male ICR mice were divided into four groups: control, restraint stress, restraint stress + MT and MT (positive control). 20 mg/kg MT or vehicle were intraperitoneally injected 60 min before restraint stress (10 h/day) once daily for 3 days. Biochemical parameters, intestinal mucosal integrity, tissues antioxidant ability and autophagic proteins levels were determined.

**Results:**

Mice subjected to restraint stress elevated NE level by 141.41% and decreased MT content by 38.82% in plasma. Consistent with the decrease in MT level, we observed a reduction in the antioxidant ability and an increase in autophagic proteins by 14.29–46.74% in the gut, resulting in injury to intestinal mucosa which was manifested by reductions in villus height and villus height/crypt depth (V/C) ratio, number of goblet and PCAN-positive cells, and expression of tight junction protein (ZO-1, occludin and claudin-1). In contrast, MT reversed these changes caused by restraint stress and improved the intestinal mucosal injury. However, there was no significant difference between MT (positive control) and control group.

**Discussion and conclusion:**

Our results suggest that MT effectively mitigates psychological stress-induced injury to intestinal mucosa, providing evidence demonstrating the potential for using MT as therapy against intestinal impairment associated with psychological stress.

## Introduction

Stress is common in modern society. A survey demonstrated that 25% of the populations of the USA are subjected to high levels of stress, while 50% of the responders reported experiencing a major stressful event during the past year (Schlegel 2009). Restraint stress was initially developed by Bonfils (1993) and has since been widely utilised to mimic psychological stress in a number of studies (Bali and Jaggi 2015; Wu et al. 2016). A growing body of evidence demonstrates that restraint stress damages the intestine and causes disorders such as inflammatory bowel disease (IBD) (Schultz et al. 2015). However, the pathogenesis underlying the induction of injury to intestinal mucosa by restraint stress remains controversial. A number of researchers have reported that gastrointestinal ulcerogenesis is caused by a deficiency in endogenous prostaglandin (PG), particularly in intestinal ulcers resulting from the use of nonsteroidal anti-inflammatory drugs (NSAID) (Adler et al. 2009). Oxidative stress was also shown to play a role in the pathogenesis of stress-induced gastric ulcers (Jia et al. 2007). Stress activates the hypothalamic-pituitary-adrenal (HPA) axis, thereby leading to excessive production of free radicals and resulting in irreversible cell damage (Mcintosh and Sapolsky 1996).

Recent studies have also shown that activation of the HPA axis causes dysregulation of gastrointestinal hormones and increases oxidative stress (Bali and Jaggi 2015). Norepinephrine (NE) is one of the primary paraventricular nucleus (PVN) neurotransmitters that regulate adrenocorticotropic hormone (ACTH) secretion during stress (Pacak et al. 1995). Despite the indications from a large number of studies that restraint stress may be a risk factor for gastrointestinal motility disorder (GIMD) and gastric stress ulcers (Zhao et al. 2014; Koh et al. 2015), the mechanisms by which restrain stress induces bowel disease remain unclear.

The activation of the HPA axis can be inhibited by melatonin (MT) (Bu and Liu 2007). At the time of discovery of MT, its binding sites were found to be widely distributed throughout the gastrointestinal tract (Ozaki and Lynch 1976). Recent studies have shown that MT exerts a multitude of effects on the digestive system, including decreasing paracellular permeability in the duodenal mucosa and increasing mucosal bicarbonate secretion (Sommansson et al. 2014), improving acid secretion in the colon, modifying the expression of several cytokines (including IL-2, IL-2R, and IFN-γ released by human CD4 T cells) (Chen et al. 2011), and reducing the force of spontaneous contractions of ileum and colon segments (Thor et al. 2007). Taken together, these effects are part of the considerable influence MT exerts on the regulation of the physiological function of the intestine.

However, the precise mechanism through which MT regulates the induction of gastric ulcers by restraint stress is not clear. A number of studies suggest that this effect of MT may be related to its ability to attenuate immunological damage caused by macrophage activity (Yi and Kim 2017), decrease restraint stress-induced oxidative stress, and inhibit the production of nitric oxide (Guo et al. 2017). Therefore, gastrointestinal hormones and oxidative stress may play a critical role in the process of GIMD pathogenesis and induction of gastric ulcers by restraint stress. Nonetheless, the mechanisms by which MT regulates intestinal mucosal injury induced by restraint stress need to be further elucidated.

## Materials and methods

### Animal procedures

Experiments in Institute of Cancer Research (ICR) male mice (8 weeks of age) were conducted in accordance with the Guide for the Care and Use of Laboratory Animals published by the Animal Welfare Committee of the Agricultural Research Organisation, China Agricultural University (Approval No. CAU20170911-2). ICR male mice (8 weeks of age) were obtained from Vital River Laboratory Animal Technology Co. Ltd. (Beijing, China) and housed under conventional conditions (25 ± 2 °C temperature; 45-60% relative humidity; 14 h per day lighting cycle with 06:30-20:30 light). Mice were allowed *ad libitum* access to water and food. After a 1-week adaptation period, 144 mice were randomly divided into four groups:

- Group 1: control group; animals were intraperitoneally injected with vehicle (0.1 mL saline containing 20 µL of absolute ethanol) 60 min before the experiment for 3 consecutive days. Food and water were taken away during the experiment time (8:00 to 18:00).
- Group 2: restraint stress group; animals were intraperitoneally injected with vehicle (0.1 mL saline containing 20 µL of absolute ethanol) 60 min before the experiment for 3 consecutive days.
- Group 3: restraint stress + MT group; animals were given intraperitoneal injections of 20 mg/kg melatonin once 60 min before restraint stress for 3 consecutive days based on previous published literature (Kolli et al. 2013; Zhang et al. 2015) and our preliminary screening.
- Group 4: MT group (positive control); animals were given intraperitoneal injections of 20 mg/kg melatonin once, 60 min before restraint stress for 3 consecutive days.

Restraint stress was administered as follows: mice were restrained in 50 mL centrifuge tubes for 10 h (from 8:00 to 18:00) After 10 h of restraint stress, mice were returned to their cages and provided accessed to food and water once daily for 3 days to simulate the sedentary behaviour in people’s daily lives. At the end of the experiment (18:00 h on the third day), all mice were euthanized under anaesthesia using 10% chloral hydrate. Plasma samples were collected and intestinal tissues (duodenum, jejunum, ileum, caecum, colon, and rectum) harvested. The experiments were repeated three separate times.

### Plasma and tissue preparations

Plasma samples were collected for the measurement of NE and MT levels. Six intestinal segments were fixed in 4% paraformaldehyde and embedded in paraffin for histological analyses. Tissue samples were rapidly frozen and stored at −80 °C for molecular analyses.

### Enzyme-linked immunosorbent assay (ELISA)

Plasma NE and MT concentrations were measured using competitive enzyme-linked immunosorbent assay (ELISA) kits (CEA908Ge for MT and CEA907Ge for NE, Uscn Life Science Inc., Wuhan, China). Detection ranges for NE and MT were 61.7–5000 ng/mL and 12.35–1000 pg/mL, respectively. All assays were performed according to the kit manufacturer’s instructions. Results were quantified by measuring optical density (OD) at 450 nm wavelength, and the concentration expressed as specific activity (ng/mL for MT and pg/mL for NE). The intra- and inter-assay variations were <10% and <12%, respectively. Six plasma samples were analysed in each group, with each sample tested in triplicate.

### Assessment of antioxidant activity and lipid peroxidation

Portions of the intestinal segments (*n* = 6) were rapidly homogenised and clarified lysates were obtained by centrifugation at 200 × *g* for 10 min at 4 °C. Tissue extracts were stored at −80 °C prior to the analysis of antioxidant activity. Protein concentration was determined using a bicinchoninic acid (BCA) kit (Beyotime, P0012). Reactive oxygen species (ROS) assay kit (CA1410, Beijing Solarbio Science & Technology Co., Ltd., Beijing, China) and five commercial kits (Nanjing Jiancheng Co. Ltd., Jiancheng, Nanjing, China) were used to assess antioxidant ability, the activities of superoxide dismutase (SOD), catalase (CAT), glutathione peroxidase (GSH-Px) activities, as well as total antioxidant capability (T-AOC), and malondialdehyde (MDA) levels were quantified using colorimetric methods. SOD, CAT, GSH-Px, T-AOC, and MDA were measured as OD at 550 nm, 405 nm, 412 nm, 520 nm, and 532 nm wavelengths, respectively. SOD, CAT, and GSH-Px activities were expressed as specific activity (units/mg protein), while T-AOC and MDA were expressed as units/mg protein and µmol/mg protein, respectively. Each sample was assayed three times. Intra- and inter-assay variations were determined to be <10%.

### Histological analyses

Paraffin-embedded sections of intestinal samples (*n* = 6) were deparaffinized and hydrated, then stained with haematoxylin and eosin (H&E) and periodic acid-Schiff (PAS). For H&E staining, a minimum of 10 tissue sections were cut from each sample and photographed at 400 × magnifications using a BX51 microscope (Olympus, Tokyo, Japan). Five longest villi in each tissue section and a total of 300 longest villi were analysed in each treatment group. Villus height (V), crypt depth (C), and the V/C ratio were measured using ImageJ software. For the analysis of PAS-staining, at least 30 random fields in 6 sections were photographed for each sample, and a total of 180 fields were analysed per treatment group. The number of goblet cells per 100 enterocytes was calculated.

For immunohistochemical staining, intestinal tissue sections were incubated with the monoclonal rabbit anti-mouse primary antibodies against claudin-1 (1:200), occludin (1:100), ZO-1 (1:200), or PCNA (1:500; Abcam, Cambridge, MA, USA) overnight at 4 °C. After a rinse in 0.01 M PBS (pH 7.4), sections were incubated with biotinylated goat anti-rabbit IgG secondary antibodies (1:200; Sigma) for 2 h at 25 °C. Following the washing, sections were incubated with streptavidin-horseradish peroxidase (1:250, Sigma) for 2 h at 25 °C. Immunoreactivity was visualised by incubating the sections in 0.01 M PBS containing 0.05% 3, 3′-diaminobenzidine tetrahydrochloride (DAB; Sigma) and 0.003% hydrogen peroxide for 10 min. Nuclei were counterstained with haematoxylin. Control sections incubated without the primary antibody were examined in all analyses for comparison. Positive cells were counted in 30 random fields from 6 cross sections of the intestine in each sample. Mean integral optical density (IOD) of positive cells was determined.

### Western blotting

Intestinal tissues (*n* = 6) were rapidly homogenised in liquid nitrogen and stored at −80 °C for western blot analyses. Total protein was extracted using a lysis buffer (62.5 mmol/L Tris-HCL, 2% SDS, 10% glycerol; Ph 6.8). After centrifugation at 12,000 × *g* for 10 min at 4 °C, supernatant was collected and protein concentration determined using a BCA kit (Beyotime, P0012). A sample of 40 µg of protein was separated using 10% SDS-PAGE and transferred onto a PVDF membrane (Millipore, Billerica, MA USA). Membranes were subsequently blocked with 5% skim milk for 2 h at room temperature. Subsequently, membranes were incubated with monoclonal rabbit anti-mouse MUC2 (1:2000, Abcam) and LC3 (1:2000, Sigma) and GAPDH (1:5000, CoWin Biotech Co., Inc.) overnight at 4 °C, followed by incubation with HRP-conjugated goat anti-rabbit antibody (1:8000, CoWin Biotech Co., Inc.) for 2 h at room temperature. Immunoblots were performed using an eECL western blot kit (CW0049; CoWin Biotech Co., Inc.). Obtained bands were scanned and analysed using Gel-Pro Analyser 4.5 software and the IOD of each band was calculated. Relative levels of MUC2 and LC3 proteins were expressed as the corresponding band IODs relative to the GAPDH bands. The results were obtained from three separate experiments.

### Statistical analysis

All results were expressed as means ± SEM and analysed using SPSS 18.0 software (PASW Statistics, Chicago, IL, USA). Experimental data were evaluated using a one-way analysis of variance (ANOVA), followed by a Tukey–Kramer *post hoc* test. Different lowercase letters represent significant differences between control, stress, and stress + MT groups. *p* < 0.05 was considered statistically significant.

## Results

### Changes in plasma levels of NE and MT

**Figure 1.**
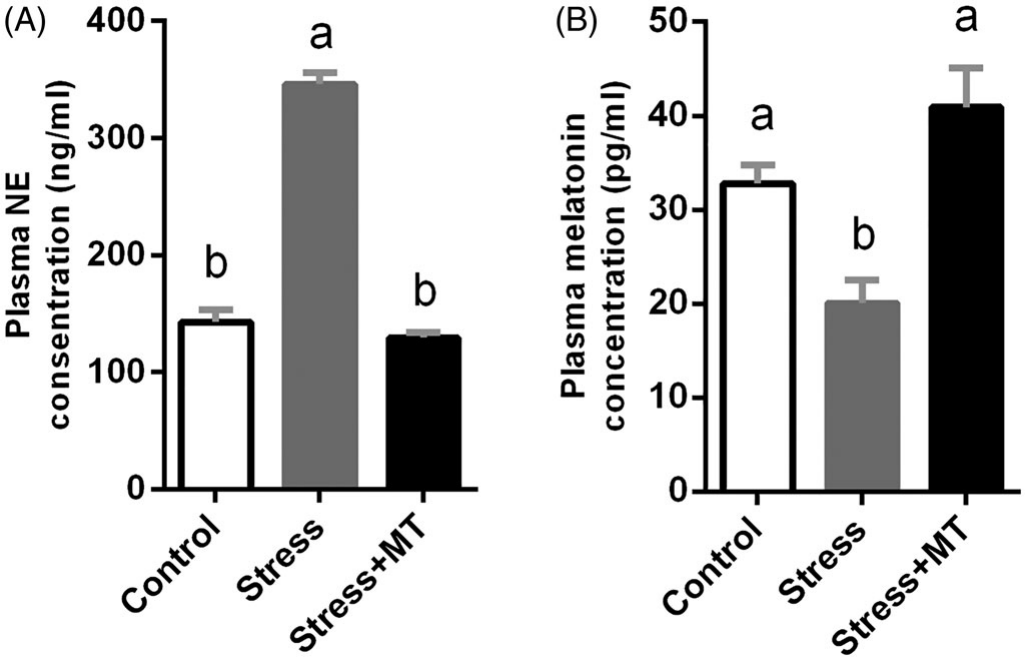
Plasma hormone levels in experimental animals: (A) effect of restraint stress and MT treatment on plasma NE concentrations; (B) effect of restraint stress and MT treatment on plasma MT concentrations. The results represent the means ± SEM for each experimental group (*n* = 6 animals). Values not sharing a common superscript letter are statistically significantly different (*p* < 0.05).

ANOVA revealed significant effects of restraint stress on plasma concentrations of NE (*F*_(2,15)_ = 167.3, *p* < 0.0001) and MT (*F*_(2,15)_ = 12.91, *p* = 0.001). As shown in Figure 1, plasma NE concentration was 141.41% higher (*p* < 0.0001) in the restraint stress group compared to that in the control group, whereas plasma MT level was 38.82% lower (*p* = 0.0347). Plasma NE levels in restrained mice were significantly lower in animals pre-treated with 20 mg/kg MT (by 62.57%, *p* < 0.0001), while MT levels were significantly higher (by 103.94%, *p* = 0.0008), relative to those of animals that received restraint stress without MT pre-treatment. However, no significant difference was observed between the control and MT-treated groups (positive control, data not shown).

### Histological changes in intestinal mucosa

**Figure 2.**
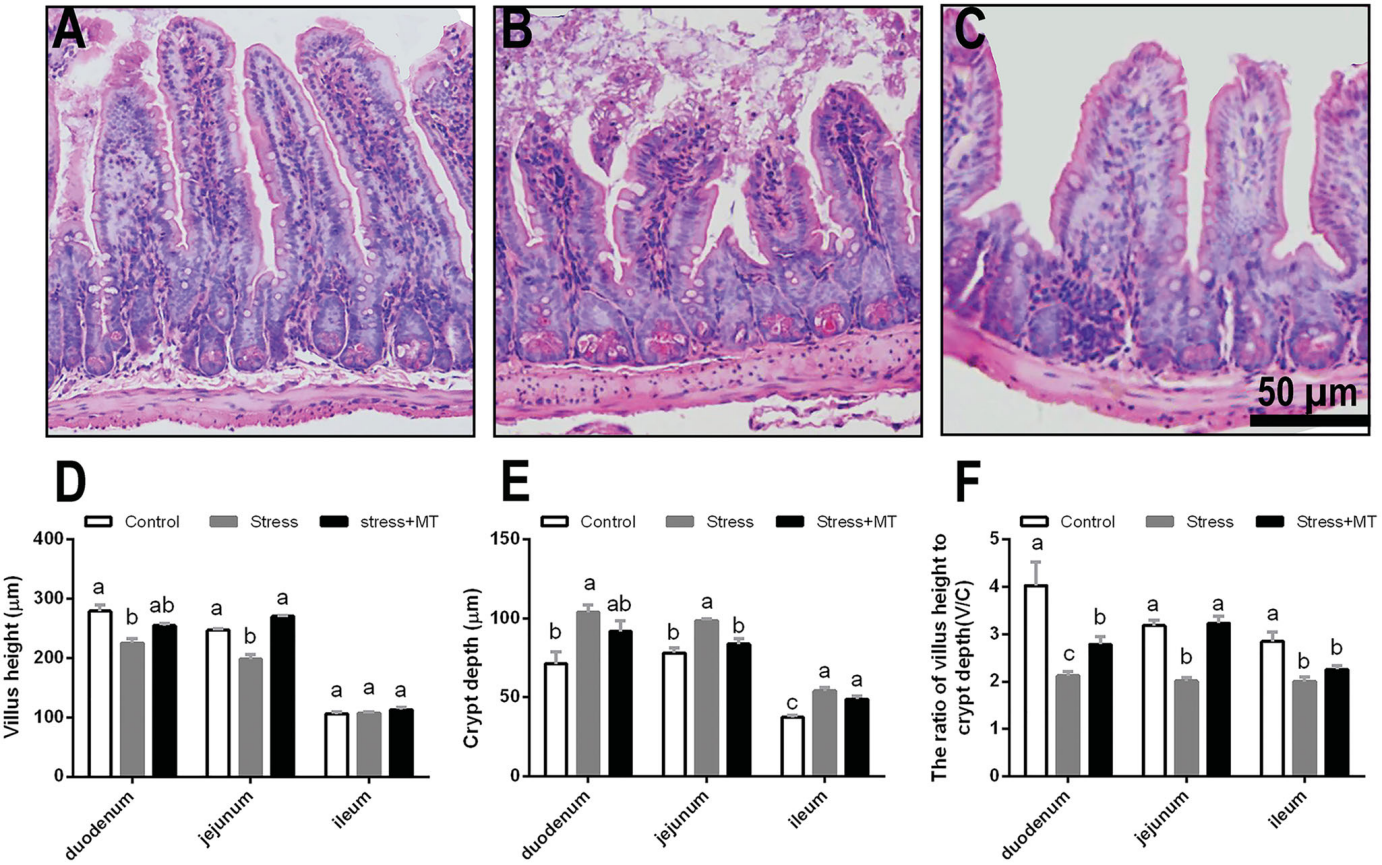
Morphology of the intestine in each experimental group was analysed using H&E staining (A–C). The following values were quantified in the images: (D) villus height; (E) crypt depth; and (F) the ratio of villus height to crypt depth (V/C). Values shown represent the means ± SEM calculated for each group (*n* = 6 animals per group). Values not sharing a common superscript letter are statistically significantly different (*p* < 0.05).

As presented in Figure 2, histological changes were detected in intestinal tissue sections by H&E staining. Animals subjected to restraint stress exhibited significant loss of crypt architecture, severe villous epithelial atrophy, and degeneration and shortening of villi. ANOVA detected significant effects of treatments on small intestinal villus height and crypt depth (*F* and *p* values presented in Table 1). In comparison with the control group, animals treated with restraint stress exhibited significantly decreased villus heights in duodenum (19.15%, *p* < 0.0001) and jejunum (21.56%, *p* < 0.0001), as well as greater crypt depth in duodenum, jejunum, and ileum (27.02–44.25%, *p* = 0.0001–0.002). Animals in the restraint stress group also had notably lower V/C ratios in the duodenum, jejunum, and ileum (29.82–46.90%, *p* = 0.0001–0.0021) compared to those of the controls. Conversely, treatment with 20 mg/kg MT attenuated the intestinal injury and resulted in the intestinal condition similar to that observed in the control group. Specifically, stress + MT treatment animals exhibited higher villus height (by 5.02–21.56%, *p* = 0.0000–0.5855), lower crypt depth (by 3.06–13.45%, *p* = 0.0000–0.5855), and higher V/C ratio (by 12.58–60.59%, *p* = 0.0000–0.4122), compared to those of animals exposed to restraint stress without MT pre-treatment. No statistically significant differences were observed between control animals and the MT-treated groups (positive control, date not shown) in these parameters.

**Table 1.** The results of ANOVA statistical tests about *F*-value and *p*-value in the various intestinal segments of three groups (control, stress and stress + MT).

Parameters	Duodenum	Jejunum	Ileum	Ileum	Colon	Colon
Villus height	*F*_(2,15)_ = 13.25, *p* = 0.0009	*F*_(2,15)_ = 58.37, *p* < 0.0001	*F*_(2,15)_ = 1.195, *p* = 0.3363			
Crypt depth	*F*_(2,15)_ = 16.98, *p* = 0.0003	*F*_(2,15)_ = 13.76, *p* = 0.0008	*F*_(2,15)_ = 19.90, *p* = 0.0002			
V/C ratio	*F*_(2,15)_ = 10.15, *p* = 0.0019	*F*_(2,15)_ = 32.88, *p* < 0.0001	*F*_(2,15)_ = 10.52, *p* = 0.0023			
Goblet cell density	*F*_(2,15)_ = 13.21, *p* = 0.0003	*F*_(2,15)_ = 12.74, *p* = 0.0002	*F*_(2,15)_ = 21.52, *p* < 0.0001	*F*_(2,15)_ = 61.63, *p* < 0.0001	*F*_(2,15)_ = 49.65, *p* < 0.0001	*F*_(2,15)_ = 76.65, *p* < 0.0001
MUC2 protein	*F*_(2,6)_ = 13.26, *p* = 0.0063	*F*_(2,6)_ = 91.52, *p* < 0.0001	*F*_(2,6)_ = 21.53, *p* = 0.0018	*F*_(2,6)_ = 18.57, *p* = 0.0027	*F*_(2,6)_ = 76.37, *p* < 0.0001	*F*_(2,6)_ = 6.804, *p* = 0.0286
ZO-1 protein	*F*_(2,15)_ = 149.5, *p* < 0.0001	*F*_(2,15)_ = 24.75, *p* < 0.0001	*F*_(2,15)_ = 8.784, *p* = 0.0034	*F*_(2,15)_ = 0.6268, *p* = 0.5477	*F*_(2,15)_ = 24.14, *p* = 0.0002	*F*_(2,15)_ = 52.50, *p* < 0.0001
Claudin-1 protein	*F*_(2,15)_ = 61.89, *p* < 0.0001	*F*_(2,15)_ = 19.70, *p* = 0.0013	*F*_(2,15)_ = 26.84, *p* = 0.0010	*F*_(2,15)_ = 230.7, *p* < 0.0001	*F*_(2,15)_ = 364.5, *p* < 0.0001	*F*_(2,15)_ = 179.9, *p* < 0.0001
Occludin protein	*F*_(2,15)_ = 55.46, *p* = 0.0001	*F*_(2,15)_ = 55.46, *p* = 0.0001	*F*_(2,15)_ = 82.74, *p* < 0.0001	*F*_(2,15)_ = 74.75, *p* < 0.0001	*F*_(2,15)_ = 898.6, *p* < 0.0001	*F*_(2,15)_ = 28.54, *p* = 0.0009
PCNA	*F*_(2,15)_ = 7.263, *p* = 0.0086	*F*_(2,15)_ = 48.82, *p* < 0.0001	*F*_(2,15)_ = 44.66, *p* < 0.0001	*F*_(2,15)_ = 35.45, *p* < 0.0001	*F*_(2,15)_ = 2.406, *p* = 0.1403	*F*_(2,15)_ = 14.59, *p* = 0.0021
LC3-I/II ratio	*F*_(2,15)_ = 66.52, *p* < 0.0001	*F*_(2,15)_ = 84.17, *p* < 0.0001	*F*_(2,15)_ = 9.451, *p* = 0.0022	*F*_(2,15)_ = 83.48, *p* < 0.0001	*F*_(2,15)_ = 22.16, *p* = 0.0007	*F*_(2,15)_ = 21.99, *p* = 0.0009
ROS	*F*_(2,15)_ =18.96, *p* = 0.0026	*F*_(2,15)_ = 12.47, *p* = 0.0073	*F*_(2,15)_ = 5.783, *p* = 0.0399	*F*_(2,15)_ = 5.239, *p* = 0.0483	*F*_(2,15)_ =5.873, *p* = 0.0386	*F*_(2,15)_ = 5.736, *p* = 0.0405
GSH-PX	*F*_(2,15)_ = 15.02, *p* = 0.0003	*F*_(2,15)_ = 3.816, *p* = 0.0457	*F*_(2,15)_ = 0.282, *p* = 0.7581	*F*_(2,15)_ = 10.20, *p* = 0.0019	*F*_(2,15)_ = 70.50, *p* < 0.0001	*F*_(2,15)_ = 6.477, *p* = 0.0094
CAT	*F*_(2,15)_ = 11.31, *p* = 0.0010	*F*_(2,15)_ = 14.47, *p* = 0.0003	*F*_(2,15)_ = 5.155, *p* = 0.0210	*F*_(2,15)_ = 87.52, *p* < 0.0001	*F*_(2,15)_ = 37.48, *p* < 0.0001	*F*_(2,15)_ = 11.04, *p* = 0.0011
SOD	*F*_(2,15)_ = 3.840, *p* = 0.0450	*F*_(2,15)_ = 20.10, *p* < 0.0001	*F*_(2,15)_ = 11.17, *p* = 0.0011	*F*_(2,15)_ = 42.72, *p* < 0.0001	*F*_(2,15)_ = 73.35, *p* < 0.0001	*F*_(2,15)_ = 15.25, *p* = 0.0002
T-AOC	*F*_(2,15)_ = 19.75, *p* = 0.0001	*F*_(2,15)_ = 9.357, *p* = 0.0023	*F*_(2,15)_ = 7.143, *p* = 0.0066	*F*_(2,15)_ = 18.43, *p* < 0.0001	*F*_(2,15)_ = 8.828, *p* = 0.0029	*F*_(2,15)_ = 231.7, *p* < 0.0001
MDA	*F*_(2,15)_ = 21.29, *p* < 0.0001	*F*_(2,15)_ = 9.701, *p* = 0.0020	*F*_(2,15)_ = 0.9691, *p* = 0.4035	*F*_(2,15)_ = 8.794, *p* = 0.0034	*F*_(2,15)_ = 8.905, *p* = 0.0032	*F*_(2,15)_ = 20.46, *p* < 0.0001

**Figure 3.**
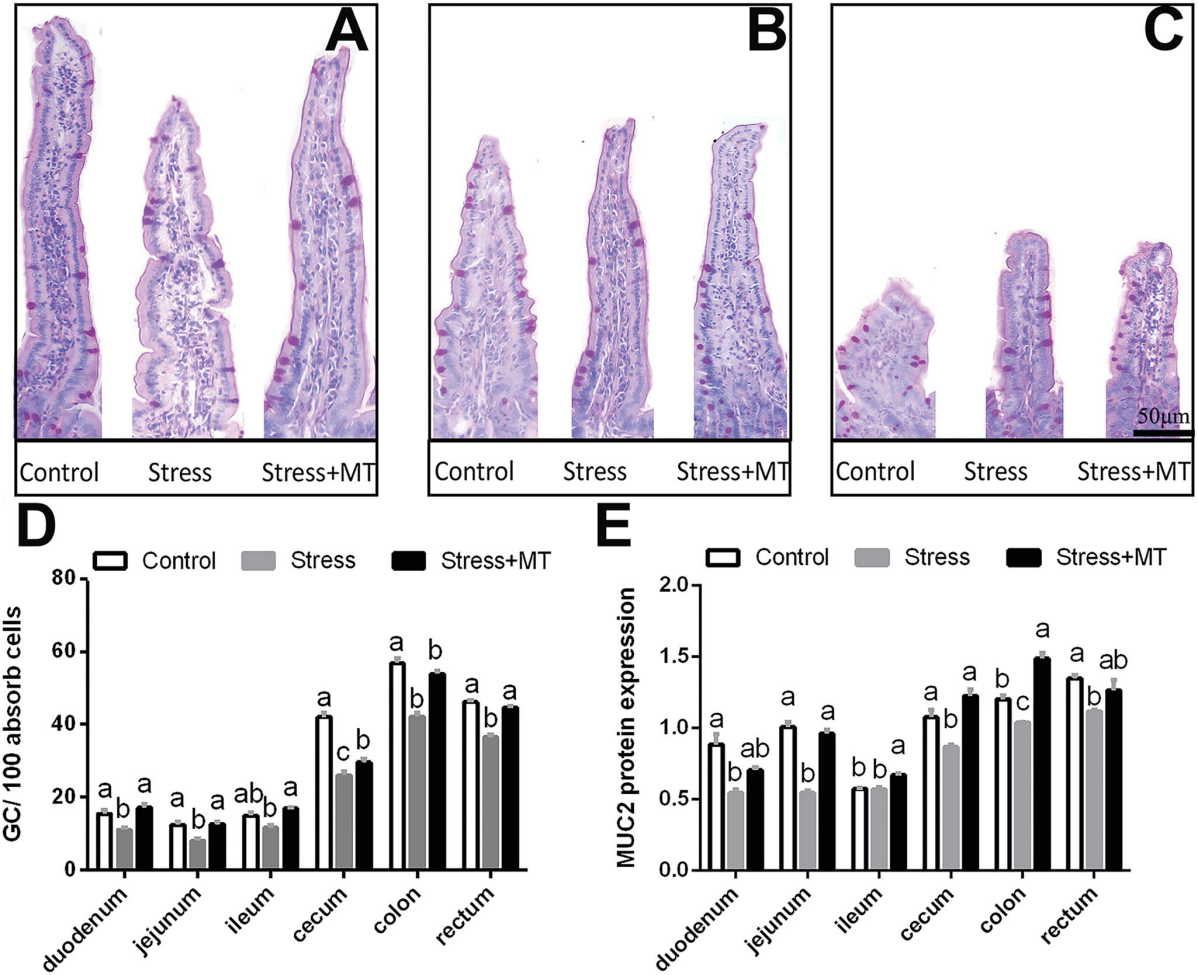
Effects of MT pre-treatment on the number of goblet cells per 100 enterocytes and the expression of MUC2 protein in mice (A–C). The number of goblet cells per 100 cells in control, stress and stress + MT experimental groups (D). Expression of MUC2 protein in treatment groups was examined by western blotting, with total protein levels normalised to GAPDH (E; *n* = 3). The results represent the mean ± SEM for each group. Differences were assessed by ANOVA, with significant difference (*p* < 0.05) observed in values which do not share a common superscript letter.

PAS staining showed that the goblet cells exhibited red staining and were dispersed among the epithelial cells, mostly in the lower half of the villi (Figure 3). Goblet cell densities were affected by restraint stress and MT treatment (Figure 3(D)). Animals subjected to restraint stress exhibited significantly lower goblet-cell densities in all intestinal segments (by 20.89–37.79%, *p* = 0.0000–0.0067), compared to the control group. A similar effect was observed on the expression of MUC2 protein, which was affected differently by the experimental treatments (Figure 3C). MUC2 protein expression was significantly lower (by 13.57–45.81%, *p* = 0.0000–0.029) in all intestinal sections of animals subjected to restraint stress compared to that of the control group, with the exception of the ileum (*p* = 0.9965). However, treatment with 20 mg/kg MT reversed the changes in goblet cell number induced by restraint stress, with 13.16–55.55% (*p* = 0.0000–0.1297) higher numbers of goblet cells found in the small and large intestines of MT-treated animals, compared with those of the restraint-stress group. Moreover, MUC2 protein expression was 13.08–75.69% (*p* = 0.0000–0.0772) higher in MT-pre-treated animals compared to that in the stress group. However, there were no significant differences between control animals and the MT-treated groups (positive control, date not shown) in these parameters.

### Changes in the expression of tight-junction proteins in intestinal epithelium

**Figure 4.**
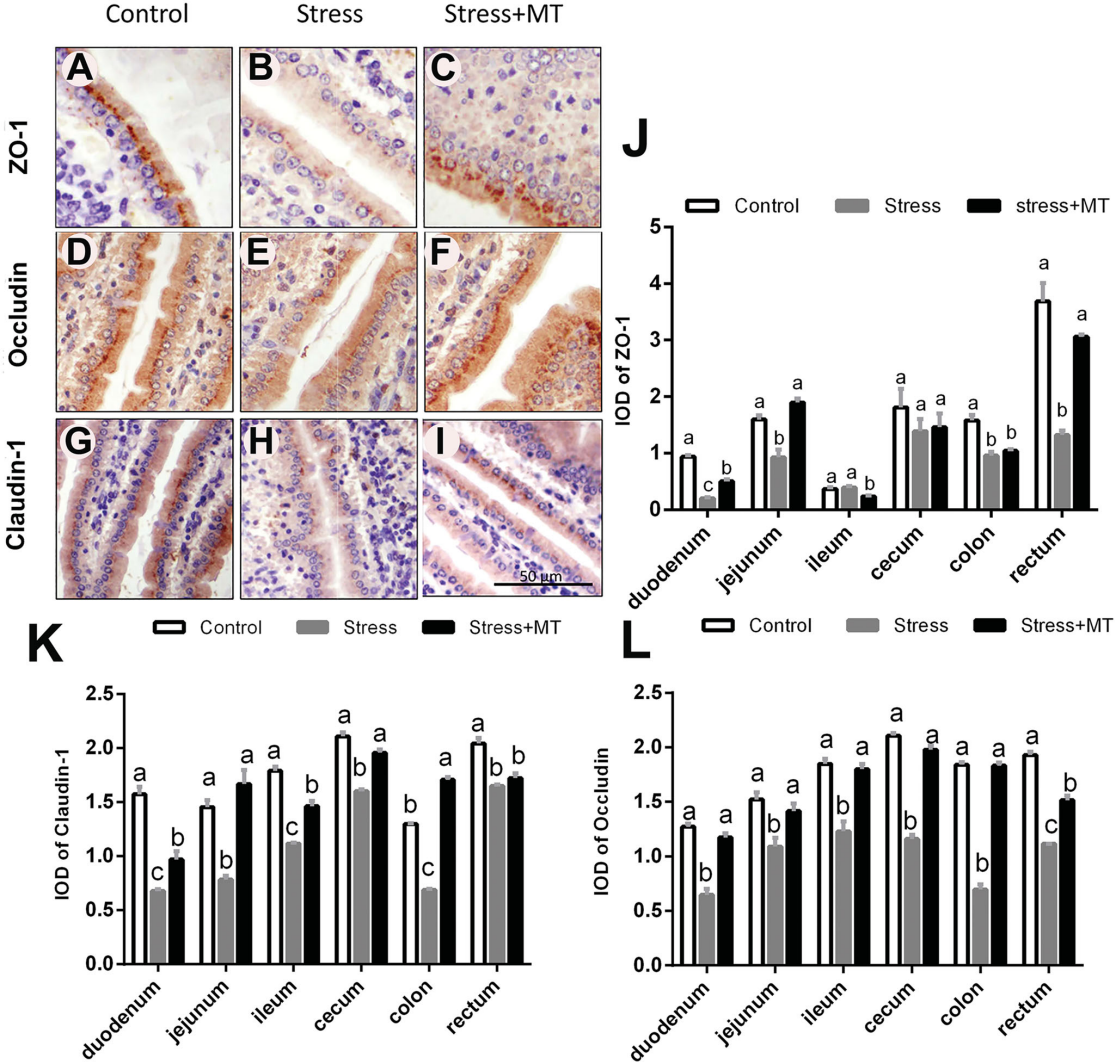
Effects of MT on tight junction proteins in mice subjected to restraint stress. Immunohistochemistry staining of ZO-1 (A–C), Occludin (D–F), and Claudin-1(G–I) in intestinal tissue sections (scale: 100 μm) obtained from animals in the Control, Stress, and stress + MT groups. IOD of ZO-1 (J), Claudin-1 (K), and Occludin (L) in the intestinal tissue from each treatment group (*n* = 6, 20 microscopic images were obtained from each treatment group). The results represent the mean ± SEM for each group. Differences were assessed by ANOVA, with significant difference (*p* < 0.05) observed in values which do not share a common superscript letter.

As shown in Figure 4, ZO-1 expression was observed along the epithelium and the vascular endothelium of lamina propria, while cells positive for occludin and claudin-1 were uniformly and continuously distributed along the epithelium and the vascular endothelium. ANOVA showed that the expression of tight junction proteins was significantly different between the treatment groups. The results show that IODs of tight junction proteins were decreased in animals subjected to restraint stress, compared with those in the control group (claudin-1, decreased by 19.27–56.96%, *p* = 0.000–0.0037; occludin-1, 28.38–62.11%, *p* = 0.000–0.0131; and ZO-1, 23.31–78.40%, *p* = 0.0000–0.5371). Animals that received MT pre-treatment prior to the restraint stress exhibited ZO-1 up-regulation (by 104.99–146.3%, *p* < 0.0001) in duodenum, jejunum, and rectum, compared to the mice subjected to restraint stress without pre-treatment. No significant difference was observed between the two groups in the ileum, caecum, and colon (*p* > 0.05). However, stress + MT treatment animals showed greater number of cells staining positive for occludin and claudin-1 proteins in all intestinal segments (29.91–162.61% higher, *p* = 0.0000–0.0431 and 4.42–147.68%, *p* = 0.0000–0.4397, respectively).

### Changes in enterocyte proliferation and autophagy

**Figure 5.**
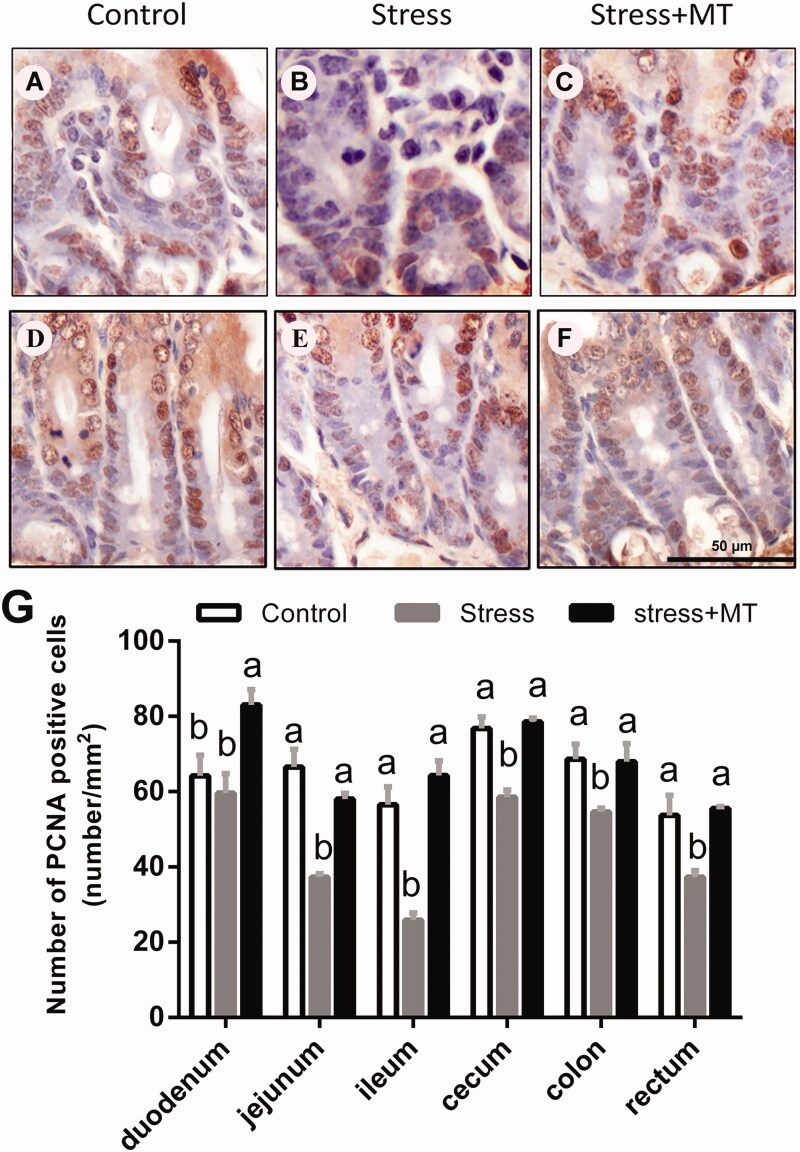
Effects of MT on the renewal ability of epithelial cell in small intestine (A–C) and large intestine segments (D–F) obtained from mice subjected to restrain stress. (G) IOD of PCNA-positive cells in intestinal tissues obtained from animals in the control, stress, and stress + MT groups (*n* = 6, 20 microscopic images were obtained from each treatment group). The results represent the mean ± SEM for each group. Differences were assessed by ANOVA, with significant difference (*p* < 0.05) observed in values which do not share a common superscript letter.

PCNA-positive cells were found scattered in tissue surrounding the intestinal glands and basal layer cells (Figure 5(A–F)). Number of PCNA-positive cells was significantly altered by restraint stress. IOD values of PCNA-positive cells were decreased by 7.68–54.22 (*p* = 0.0001–0.0676) in intestinal segments of animals in the restraint-stress group compared to those in the control animals (Figure 5(G)). However, stress + MT treatment group exhibited higher PCNA IODs (by 39.27–148.14%, *p* = 0.0001–0.0098) in the intestinal segments, as compared to that of the restraint stress group.

**Figure 6.**
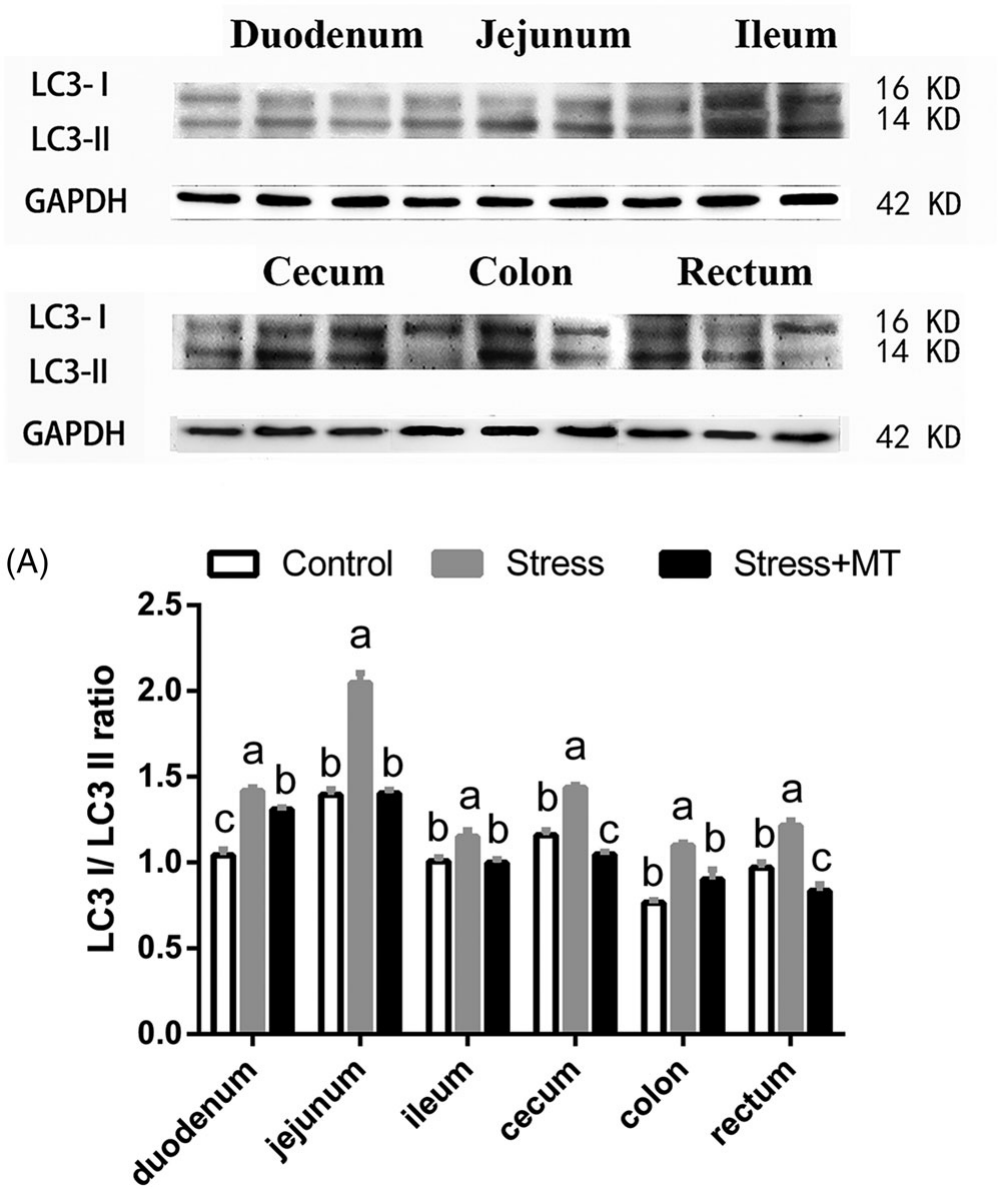
Effects of MT on the expression of autophagy proteins in mice subjected to restraint stress. The protein sizes of LC3-I, LC3-II, and GAPDH were 16, 14, and 42 KD, respectively, (A) LC3-I/II protein expression levels in intestinal tissues of animals in the control, stress, and stress + MT groups, normalised to GAPDH expression (*n* = 6 for each protein). The results represent the mean ± SEM for each group. Differences were assessed by ANOVA, with significant difference (*p* < 0.05) observed in values which do not share a common superscript letter.

Expression of autophagic protein LC3 was assessed in the intestine by western blot (Figure 6). LC3-I/II ratio was found to be significantly different between the treatment groups (*F* and *p* values presented in Table 1). Following the induction of restraint stress, LC3-I/II ratio was increased (by 14.29–46.74%, *p* = 0.0000–0.0062), compared to that of the control group. However, treatment with 20 mg/kg MT attenuated the upregulation of autophagy proteins by the restraint stress. Specifically, the LC3 I/II ratio in MT-treated animals was 7.55–31.46% lower (*p* = 0.0000–0.015) relative to the restraint-stress group.

### Changes in antioxidant capacity in intestinal tissue

**Figure 7.**
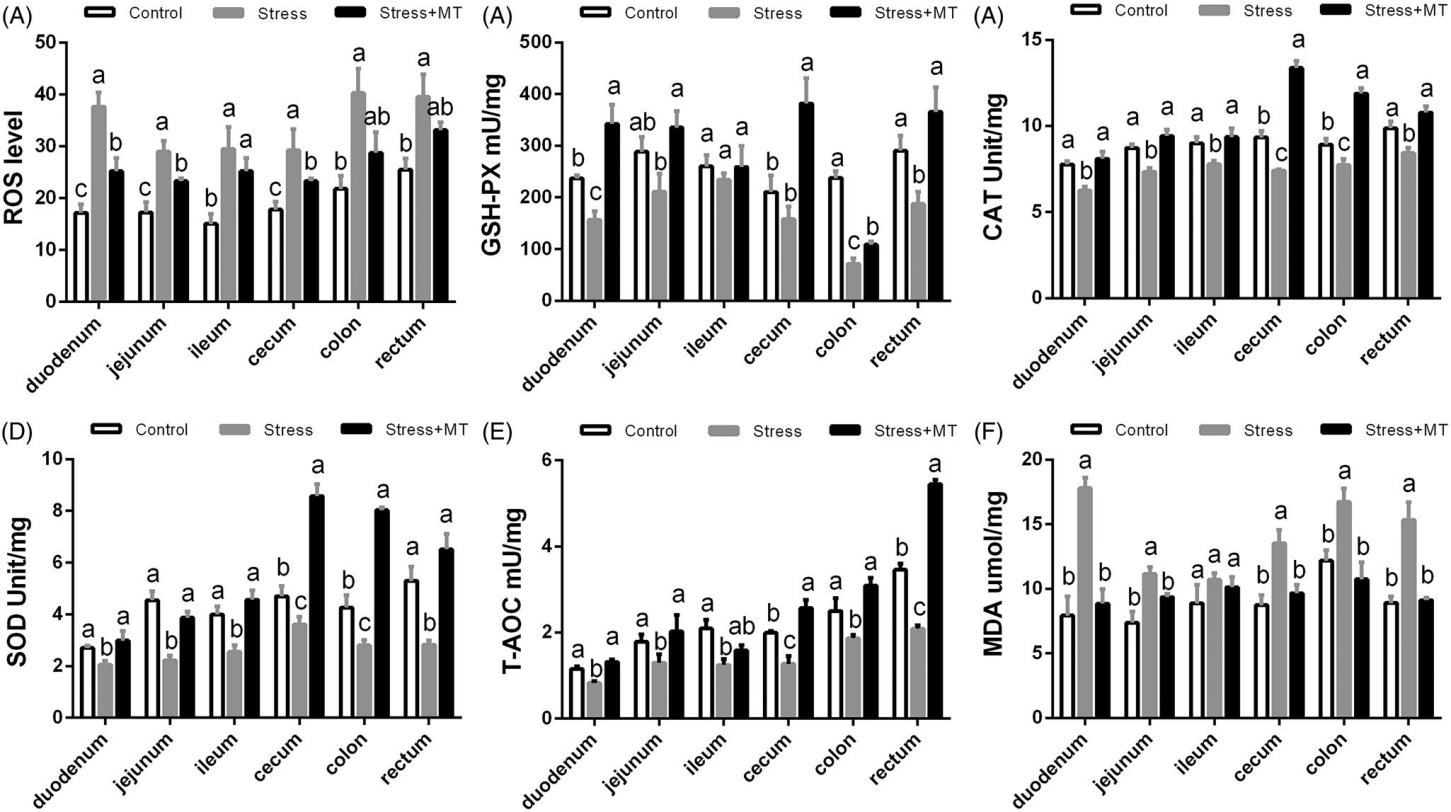
Effects of MT on antioxidant parameters in mice subjected to restraint stress. ROS levels (A), and GSH-PX (B), CAT (C), SOD (D), T-AOC (E), and MDA (F) activities in intestinal tissues were measured by tests for enzymes involved in oxidative stress. The results represent the mean ± SEM for each group (*n* = 6 animals). Values not sharing a common superscript letter are statistically significantly different (*p* < 0.05).

We further examined the changes in antioxidant capacity in the intestinal tissues. As assessed by ANOVA, ROS levels, as well as activities of antioxidant enzyme GSH-PX, CAT, and SOD, were significantly different between the treatment groups. In animals subjected to restraint stress, ROS were 55.17–119.37% higher (*p* = 0.0021–0.0407), while antioxidant enzyme activity of GSH-PX, SOD, and CAT were reduced by 10.82-69.74% (*p* = 0.000–0.7841), 13.36–46.51% (*p* = 0.0067–0.1374), and 13.26 –20.64% (*p* = 0.0016–0.0705), respectively, compared to those of the control animals. Similarly, restraint stress resulted in a 25.44–40.46% decrease in T-AOC levels (Figure 7(E); *p* = 0.000–0.1043) compared to that in the control group. Consistent with the changes in ROS and antioxidant enzyme activities, MDA content was elevated (by 20.56–124.5 9%, *p* = 0.0000–0.3806, Figure 7(F)) in the restraint stress group compared to that in the control group. In contrast, animals which underwent pre-treatment with MT exhibited an attenuation in the stimulatory effects of restraint stress on ROS levels, antioxidant enzyme activity, and lipid peroxidation, with GSH-PX, SOD, CAT, NOS, T-AOC, and MDA levels returned to the levels not statistically different from those of the control group (*p* > 0.050), or even showed an improvement in some intestinal tissues.

## Discussion

In the present study, we found that restraint stress significantly increased plasma levels of NE (by 141.41%) and corticosterone (data not shown), compared to the control group. This finding corroborates the results of past studies in which exposure of rats to 30 min of restraint stress increased hypothalamic NE concentration, while longer restraint was associated with an attenuation of NE upregulation (Keim and Sigg 1976). HPA axis activation is recognised as a key component of the physiological response to stress (Adinoff et al. 2005). Additionally, decreased plasma MT levels (by 52.57%) were observed in parallel with increased plasma NE levels following restraint stress, informing our hypothesis that MT may be involved in the response to short-term restraint stress. We therefore assessed the effect of exogenous MT supplementation in mice subjected to restraint stress, with 20 mg/kg MT administered by intraperitoneal injection 1 h before the beginning of daily restraint stress in stress + MT group and MT treatment group (positive control). The supplementation time and dose were selected on the basis of published studies and our preliminary experiments. In past studies, 15 mg/kg MT administered by intraperitoneal injection 30 min before noise stress was observed to reduce the induction of GIMD (Zhang et al. 2015), while intraperitoneal injection of 40 mg/kg effectively attenuated methotrexate-induced oxidative stress and small intestinal damage (Kolli et al. 2013). Our results showed that intraperitoneal injection of 20 mg/kg MT to mice subjected to restraint stress reversed the reduction in plasma MT levels following restraint stress and returned MT levels to normal levels. Therefore, the selected dose and route of MT supplementation were expected to be effective in mice treated by restraint stress. Moreover, in parallel with the elevation in plasma MT level, plasma NE concentration in mice exposed to restraint stress was reduced to the levels of the control group. However, there were no significant changes between MT treatment group (positive) and control groups. These results indicate that intraperitoneal injection 20 mg/kg MT to mice without restraint stress have no changes on the plasma biochemical parameters, which indicated that the restraint-stress mouse model was successfully implemented with and without MT supplementation in this study.

Pathogenesis of stress-induced intestinal mucosal injury is a complex process, with parallels to the effects of NSAID overdoses (Adler et al. 2009), sleep deprivation (Gao et al. 2019), and excessive alcohol consumption (Chang et al. 2013). Using the described animal model, we evaluated whether restraint stress can induce intestinal mucosal injury. We found that restraint stress significantly damaged intestinal mucosa in mice, as manifested through the observed increases in crypt depth and decreases in mean villus height, V/C ratios, and goblet cell numbers, as well as reductions in the expression of MUC2 and tight-junction proteins (ZO-1, occludin and claudin-1). Consistent with the present study, damage to the gut morphological integrity was previously shown to be enhanced in the jejunum, ileum, and colon of mice by chronic restraint stress (Cameron and Perdue 2005). Several studies have demonstrated that intestinal villus structure represents the most important factor in the physiological function of the intestine, with a key role in digestion/absorption and barrier functions (Nalle and Turner 2015; Salvo Romero et al. 2015). Moreover, goblet cells secrete antimicrobial peptides and mucus to maintain the composition of the gut microbiome (Heazlewood et al. 2008). Mucus secretion and mucus layer formation form the first layer of intestinal mucosal barrier (Van et al. 2006). Additionally, tight junction proteins form a structure at the boundary of adjacent cells, working as a selectively permeable barrier within the epithelial cell space (Acharya et al. 2004). Taken together, our understanding of the intestinal layers suggests that restraint stress could relatively quickly lead to damage to the intestinal mucosa, disrupting the mechanical barrier and adversely affecting digestion and absorption functions.

In our current study, we analysed the mechanisms by which restraint stress influences mucosal injury. We found that restraint stress decreased the number of PCNA-positive cells and increased the LC3-I/LC3-II ratio in the intestinal epithelium compared to the control group. It is generally accepted that, under most biological conditions, autophagy functions as a pro-survival mechanism with modest activation, as measured by LC3 conjugation (Mathew et al. 2007). Additionally, autophagy may be a mechanism of cell death under specific conditions, participating in a process called ‘autophagic cell death’ through poorly understood mechanisms (Kroemer et al. 2010). Conversely, PCNA protein is central to both DNA repair and replication and is expressed exclusively in proliferating normal cells. One of the well-established functions of PCNA is its role as the processivity factor for DNA polymerase delta and epsilon. Down regulation of PCNA is an indication of decreased cell proliferation. Therefore, dysfunction of the intestinal barrier through a downregulation of cell proliferation and upregulation of cell autophagy may cause a decrease in enterocyte renewal and result in uncontrolled flow of antigens across the intestinal epithelium, in turn challenging the immune system and affecting the host microbial balance, initiating inflammatory mechanisms, and affecting distant organ systems (Meddings 2008; Yu et al. 2012). The findings of the present study suggest that alterations in autophagic activity in intestinal tissues and a down regulation of cell proliferation occur in response to intestinal mucosal injury induced by restraint stress.

Interestingly, a decrease in plasma MT levels was detected in restraint stress-induced intestinal mucosal injury, which led us to hypothesise that MT may be involved in the modulation of intestinal mucosal integrity. To investigate this effect further, we evaluated a group of animals receiving exogenous MT supplementation (stress + MT group). Our results suggest that MT administration can reverse intestinal mucosal injury caused by restraint stress, in parallel with an elevation in plasma MT levels. MT pre-treatment also resulted in attenuation of restraint stress-induced damage to intestinal mucosal structure, including an increase in villus height, V/C ratio, goblet cell number, expression of MUC2 and tight-junction proteins, and cell proliferation, as well as a decrease in cell autophagy. Our results corroborate the reports of previous studies, showing that intraperitoneally injected MT can attenuate noise stress-induced gastrointestinal motility disorder and gastric stress ulcer in rat (Zhang et al. 2015). MT also alleviates intestinal damage induced by weaning stress in mice by improving body weight gain, intestinal morphology, and the composition of intestinal microbiota (Ren et al. 2018). Taken together with the past studies, our results suggest that MT can prevent the induction of intestinal mucosal damage by restraint stress and accelerate intestinal mucosa renewal.

The mechanisms by which MT exerts its protective effects and alleviates restraint stress-induced injury to the intestinal mucosa remain to be explained. MT is known to be an endogenous indoleamine, produced and secreted by the pineal gland. A large number of studies have demonstrated that MT plays an important role in antioxidant and anti-inflammatory processes (Trivedi and Jena 2013; Trivedi et al. 2016). In the present study, we found that restraint stress decreased plasma MT levels, in parallel with a decrease in intestinal antioxidant capacity. More specifically, intestinal ROS levels were increased following restraint stress, while the activity of antioxidant enzymes SOD, CAT, and GSH were significantly decreased, lead resulting in decreased levels of T-AOC and increased levels of MDA. Previous studies have shown that exposure to stressful conditions leads to excessive production of free radicals, causing an imbalance in the oxidant/antioxidant system (Groot et al. 2006). Recently, oxidative and nitrosative stress have been aetiologically implicated in a wide variety of disease processes and states: aging, ischemia-reperfusion injury, hypertension, atherosclerosis, diabetic neuropathy, renal diseases, neurological diseases (including Alzheimer’s disease and other forms of dementia), and cancer (Faraci 2005; Butterfield et al. 2007; Hamel et al. 2008). Additionally, oxidative stress is one of the major factors capable of disrupting the intestinal epithelial barrier function (Kekec et al. 2009). A recent study has shown that heat stress-induced severe oxidative stress in pig intestinal tissues, which consequently affected intestinal integrity (Pearce et al. 2013). Conversely, antioxidant enzymes are known to protect intestinal cells against oxidative damage (Ozkan et al. 2009). In our current work, levels of antioxidant enzymes (SOD, CAT, and GSH) were recovered almost to the normal levels after treatment with MT. Consistent with the changes in antioxidant system in the mouse intestine, damaging to the intestinal mucosa induced by restraint stress was reduced in animals pre-treated with MT. Therefore, MT may alleviate restraint stress-induced intestinal injury by relieving oxidative stress.

In summary, our results provide original data showing that acute restraint stress exerts a damaging effect on the mouse intestine, primarily through the induction of oxidant stress, elevation of ROS levels, stimulation of autophagy, and reduction of epithelial cell proliferation, resulting in damage to the intestinal mucosal barrier. In contrast, pre-treatment with 20 mg/kg MT reversed the changes caused by restraint stress and reduced the intestinal mucosal injury. Our results suggest that MT may be effective in mitigating psychological stress-induced intestinal mucosal injury. Based on these findings, we propose that MT should be considered for use as a novel therapy for stress-induced intestinal dysfunction.

## Author contributions

Y.C and R.L. contributed to the study design; Y.C. obtained funding; R.L. performed the experiments; Z.W., J.C. and T.G. analysed the data; Y.C., R.L. and Y.D. wrote the manuscript. All authors reviewed the final manuscript.
